# Clinical Inertia in Urgent Dialysis Initiation: Understanding and Overcoming Barriers to Change

**DOI:** 10.34067/KID.0000000937

**Published:** 2025-08-28

**Authors:** Megha Salani, Thomas Golper

**Affiliations:** 1 Division of Nephrology and Hypertension, Vanderbilt University Medical Center, Nashville, Tennessee; 2 Vermont University Medical Center, Burlington, Vermont

**Keywords:** dialysis

## Introduction

In the setting of an urgent need to start dialysis, clinical inertia often leads to the initiation of hemodialysis, regardless of which dialysis modality is the most appropriate for the patient. To better align treatment with patient preferences, we must understand the factors that contribute to this inertia and the steps needed to overcome it.

Consider a patient admitted with signs and symptoms of kidney failure. The patient has not engaged in routine health care visits and is not familiar with dialysis. He is eager to recover so that he can go back to work full-time and spend evenings with his family. Peritoneal dialysis (PD) may be the most suitable option to maintain his desired quality of life. This discussion will explore potential barriers to this patient starting on PD and opportunities to address them.

## Provider Concerns

Providers may perceive hemodialysis to be more effective than PD in this acute setting. However, the evidence does not support this view. Multiple studies, including a retrospective analysis in Finland and two randomized controlled trials in Thailand and China, found similar patient survival on urgent-start PD compared with urgent-start hemodialysis, with fewer complications in the PD group.^1–3^

In this issue of *Kidney360*, García-García *et al*. demonstrate the safety and efficacy of urgent-start PD compared with hemodialysis in patients with severe and symptomatic kidney failure.^4^ Because this prospective study only included patients with severe azotemia, the cohort had a higher mortality rate than previously reported. However, like the aforementioned studies, the mortality rates were similar for patients on both hemodialysis and PD. Patients in each group had similar baseline rates of volume overload and hyperkalemia and similar improvements in their metabolic parameters. These findings are significant because they can allay concerns about using urgent-start PD in patients with very advanced uremia in institutions where PD catheters can be placed as quickly as hemodialysis catheters.

Providers may defer in-depth discussions about dialysis modalities to the outpatient setting. A retrospective cohort study at the University of Rochester between January 2004 and September 2009 found that out of 124 patients who opted for PD after dialysis education, only 59 started on PD and only 60 patients were on PD at day 91.^5^ This portends a low likelihood of transition to PD for our patient if he is discharged on hemodialysis. Several factors can influence this decision: emotional overwhelm, perceived complexity of home dialysis, or negative perceptions of PD due to anecdotes of complications. In addition, on review of the Canadian Organ Replacement Register between 2001 and 2010, Nessim *et al*. found that the risk of technique failure was higher in patients who switched to PD from hemodialysis compared with those who started on PD, with an adjusted hazard ratio of 1.37.^6^ Although this is not meant to discourage transfers from hemodialysis to PD, such studies elucidate the importance of the time and effort spent to initiate patients on PD who prefer to perform PD. García-García *et al.* demonstrated that this approach is feasible even in patients with advanced uremic symptoms.^4^

Providers may lack familiarity with prescribing urgent-start PD. At Vanderbilt University Medical Center, we protocolized our inpatient urgent-start PD program in 2019 to ensure safe and effective PD initiation. The protocol includes preoperative bowel regimens, fill volume suggestions, and guidance on cycler duration and exchange frequency based on clinical status. Low fill volumes of 700 ml for women and 1000 ml for men (500–750 ml if body surface area ≤1.7 m^2^), while supine minimizes the risk of leaks; volumes are lowered if leaks occur.

## Patient Factors

After the shock of the kidney failure diagnosis, participating in dialysis decisions can be challenging. The uremic milieu can affect comprehension and retention of information. Even patients with prior dialysis, education may not recall that information at the time of initiation, and they may have had changes in their health or support systems that affect the decision for appropriate KRT.

A shared decision-making process is often possible before access placement. A multifaceted approach can balance the need for urgent decision making with the time needed to educate patients and their care partners to make the best decision. At Vanderbilt University Medical Center, we discuss options for initial modality and explain that this does not preclude other options in the future. We provide informational folders that include online resources for continued learning. Our knowledgeable team, including an inpatient nurse practitioner, case manager, and outpatient kidney care advocate, offers education, answers questions, and facilitates transitions from inpatient to outpatient settings.

Even with fill volumes lower than typical outpatient PD prescriptions, urgent-start PD in the hospital for 2–3 days provides sufficient clearance of uremic toxins to improve symptoms. Postdischarge, fill volumes can be gradually increased, and dialysis may be extended beyond typical training hours for more clearance. Care partners, if present, can initially be more hands-on, with patients trained or retrained as symptoms improve.

## Systemic Barriers

In the United States, for Medicare-eligible patients without insurance, Medicare is retroactive to the start of the month in which home dialysis is initiated. However, PD may not be an option for patients without access to insurance coverage. A prospective cohort study done in Saudi Arabia from May 2021 to June 2023 illustrated a considerable cost difference with dialysis modality. The monthly cost for PD was $2652 compared with $6590 for in-center hemodialysis.^7^ This monthly cost difference is similar in the United States, owing to staffing, consumable, and overhead costs as well as external services. A cost analysis of emergency department visits and hemodialysis sessions for uninsured patients might reveal savings if funding were directed to PD, potentially improving quality of life for patients able to dialyze at home.

The facility may lack the infrastructure for urgent-start PD. To have a successful program, an institution must invest time, energy, money, and space. Above, we discussed the cost savings that would make this investment worthwhile. For hospitals that do not have outpatient dialysis units, there are still cost savings. For example, patients who undergo urgent-start PD and hemodialysis have similar rates of infections.^1–3^ However, peritonitis can often be treated at home with intraperitoneal antibiotics. A hemodialysis catheter-related infection typically requires rehospitalization, intravenous antibiotics, catheter removal, and catheter replacement, costing $17,000–$32,000 per episode.^8^

Timely placement of PD catheters is key to a successful urgent-start PD program. Appropriate infrastructure includes commitment from trained surgeons, interventional radiologists, or nephrologists to place catheters within 48 hours. In situations where catheter placement is delayed and medical management is not sufficient, temporary hemodialysis may be needed. In centers where PD is delayed due to catheter placement, interventional radiology may be an underutilized resource.^9^

Globally, there are issues with workforce shortages within nephrology at a time of increasing prevalence of kidney disease. Workforce shortages can limit patients' access to care and increase demands on health care providers. These in turn lead to more suboptimal dialysis starts and places further demand on the system. These issues need to be addressed to create a more sustainable system.

## Conclusion

Addressing the factors that contribute to clinical inertia in urgent dialysis initiation is vital to optimize patient care. Urgent-start PD is a safe and effective treatment for kidney failure. It allows patients to start on PD first, avoiding hemodialysis catheters and the associated risk of bloodstream infections. Starting patients on hemodialysis in acute settings due to clinical inertia decreases PD uptake and increases the risk of technique failure in those who do transfer to PD. Strategies for change include provider education and protocol development, patient education and advocacy, and systemic changes to improve access to care and workforce development.
